# A potential treatment of COVID-19 with TGF-β blockade

**DOI:** 10.7150/ijbs.46891

**Published:** 2020-04-21

**Authors:** WanJun Chen

**Affiliations:** Mucosal Immunology Section, NIDCR, National Institutes of Health, Bethesda, MD 202892, USA

The rapid spread of coronavirus SARS-CoV-2 causing COVID-19 across the world poses a major threat to the global health. The major cause of death in infected patients is severe acute respiratory distress syndrome (ARDS) caused by the SARS-Cov-2 virus. Similar to the SARS virus and unlike the seasonal influenza virus, the high mortality rate in COVID-19 patients without visible immunodeficiency has caused panic in the general population. The current lack of specific and effective therapies for the COVID-19 is one of the major problems in controlling disease severity. Based on the clinical and laboratory features and lung pathological manifestations of COVID-19 patients combined with published pathological and immunological features present in the lungs of previous SARS patients, I hereby propose a potential immunotherapy for the severe COVID-19 through blockade of transforming growth factor-beta (TGF-β).

## Rational and hypothesis

In the majority of patients with COVID-19, death has been caused by lung failure due to severe acute respiratory distress syndrome. The syndrome is attributed to largely uncontrolled inflammatory responses characterized by a “cytokine storm”, and edema and fibrosis in the lungs at the end stages. The fibrosis in the lung may be caused mainly by transforming growth factor-beta (TGF-β). In addition, TGF-β is involved in the fluid homeostasis in the lung as well. This leads to the functional failure of the lungs and death of the patients. The massive increase in active TGF-β in the lungs, may be the result of at least three possible sources:

1), SARS-CoV-2 virus infection and consequent strong immune and inflammatory responses as well as dysregulation of the coagulation and fibrinolytic pathways induce massive activation of the latent (inactive) TGF-β in the lungs as well as latent TGF-β pool in the blood of patients. Thus, a decrease in circulating levels of total (latent) TGF-β is expected in patients with all stages of pneumonia, especially from mild to severe stage of pneumonia; at the same time, more active TGF-β in the lungs may be observed;

2), SARS-CoV-2 virus infection induces massive increases of neutrophil infiltration into the lungs where, the neutrophils can release stored TGF-β that can be activated by elastase in neutrophils. TGF-β itself can be a potent chemokine-like molecule that recruits more neutrophils into lungs to form a positive feedback loop, which can contribute to local increases in total TGF-β release and TGF-β activation;

3), SARS-CoV-2 virus infection causes apoptosis of bronchial epithelial cells, pneumocytes, and T lymphocytes. The virus can also result in the death of neutrophils. To clear the battlefield, more macrophages migrate and infiltrate into the lungs, where they engulf and digest these apoptotic cells. This consequently produces and secretes large amounts of latent (and active) TGF-β into the lungs. The produced latent TGF-β can be further activated by local proteases such as furin, plasmin and elastase, reactive oxygen species (ROS), Matrix metalloproteinases (MMPs), and integrins such as αVβ6.

As a result, the sudden and uncontrolled increases in active TGF-β (possibly with the help of some proinflammatory cytokines such as TNFα, IL-6, and IL-1β) inevitably result in rapid and massive edema and fibrosis that remodels and ultimately blocks the airways. This leads to the functional failure of the lungs and death of the patients.

## Proposed Therapy

Blockade of TGF-β function by neutralization and elimination of excess active TGF-β with anti-active TGF-β antibodies and/or TGF-β inhibitors in COVID-19 patients.

## Therapeutic goal

Prevent and block the development of fibrosis in the lungs to protect the function of the lungs and save the life of the patients.

## Indications for the therapy

*1), Symptoms:* Appearance of dyspnea and other symptoms of pneumonia.

*2), CT/X-ray manifestations of the lungs****:***Appearance of multiple small plaques and interstitial changes in the lung periphery; Deterioration to bilateral multiple ground-glass opacity and/or infiltrate shadows.

*3), Laboratory parameters:* Decreases in total (latent) TGF-β amounts in patient plasma, and/or increases in total and active TGF-β in respiratory secretions, if possible.

## Design of the clinical trials

*1), Control group:* Regular anti-viral and non-specific supportive treatment.

*2), Single treatment group:* Regular anti-viral and non-specific supportive treatment plus injection of anti-active TGF-β1,(2,3) antibody.

*3), Combined treatment group:* Regular anti-viral and non-specific supportive treatment plus administration of anti-active TGF-β1,(2,3) antibody (same as group b) plus antibodies against other proinflammatory cytokines (e.g. TNFα, IL-1β and IL-6 ).

## Phases of clinical trials

I: Safety; II: Effectiveness; III: Large number of patients.

## Endpoint and evaluation of the therapy

1),* Stop* if there is no improvement and amelioration of symptoms in patients after treatment;

2), *Stop immediately* if the symptoms of the patients worsen after treatment.

3), *Stop* if there are any signs of increased inflammatory cytokines in the blood after treatment.

4), *Consider stopping* if the patient symptoms disappear and lung bilateral multiple ground-glass opacity and/or infiltrate shadows disappear, and lungs are cleared.

## Preclinical animal study

If there are proper animal models of COVID-19, the aforementioned TGF-β blocking therapy should be tested in animals first before going to clinical setting.

